# Pluronic^®^ F127 Hydrogel Containing Silver Nanoparticles in Skin Burn Regeneration: An Experimental Approach from Fundamental to Translational Research

**DOI:** 10.3390/gels9030200

**Published:** 2023-03-06

**Authors:** Pedro Francisco, Mariana Neves Amaral, Afonso Neves, Tânia Ferreira-Gonçalves, Ana S. Viana, José Catarino, Pedro Faísca, Sandra Simões, João Perdigão, Adília J. Charmier, M. Manuela Gaspar, Catarina Pinto Reis

**Affiliations:** 1 Research Institute for Medicines (iMed.ULisboa), Faculty of Pharmacy, Universidade de Lisboa, 1649-003 Lisbon, Portugal; 2 Instituto de Biofísica e Engenharia Biomédica (IBEB), Faculdade de Ciências, Universidade de Lisboa, Campo Grande, 1749-016 Lisbon, Portugal; 3 Centro de Química Estrutural, Institute of Molecular Sciences, Faculdade de Ciências, Universidade de Lisboa, 1749-016 Lisbon, Portugal; 4 Faculdade de Medicina Veterinária, Universidade Lusoófona de Humanidades e Tecnologias, 1749-024 Lisbon, Portugal; 5 CBIOS—Research Center for Biosciences & Health Technologies, Universidade Lusófona de Humanidades e Tecnologias, Campo Grande 376, 1749-024 Lisbon, Portugal; 6 DREAMS, Universidade Lusófona de Humanidades e Tecnologias, Campo Grande 376, 1749-024 Lisbon, Portugal

**Keywords:** silver nanoparticles, burns, wound healing, nanotechnology, topical administration

## Abstract

Presently, skin burns are considered one of the main public health problems and lack therapeutic options. In recent years, silver nanoparticles (AgNPs) have been widely studied, playing an increasingly important role in wound healing due to their antibacterial activity. This work is focused on the production and characterization of AgNPs loaded in a Pluronic^®^ F127 hydrogel, as well as assessing its antimicrobial and wound-healing potential. Pluronic^®^ F127 has been extensively explored for therapeutic applications mainly due to its appealing properties. The developed AgNPs had an average size of 48.04 ± 14.87 nm (when prepared by method C) and a negative surface charge. Macroscopically, the AgNPs solution presented a translucent yellow coloration with a characteristic absorption peak at 407 nm. Microscopically, the AgNPs presented a multiform morphology with small sizes (~50 nm). Skin permeation studies revealed that no AgNPs permeated the skin after 24 h. AgNPs further demonstrated antimicrobial activity against different bacterial species predominant in burns. A chemical burn model was developed to perform preliminary in vivo assays and the results showed that the performance of the developed AgNPs loaded in hydrogel, with smaller silver dose, was comparable with a commercial silver cream using higher doses. In conclusion, hydrogel-loaded AgNPs is potentially an important resource in the treatment of skin burns due to their proven efficacy by topical administration.

## 1. Introduction

Skin is responsible for a very different set of functions essential for human survival, including acting as a barrier [1,2,3,4,5]. It is composed of three main layers, the epidermis, dermis and hypodermis, differing in composition and purpose [2,3]. Upon injury, the skin undergoes wound healing, an intricate and dynamic physiological process through which the skin repairs itself, a key process to restore its normal function [6]. To heal, the wound will undergo four stages. The first stage is haemostasias, starting with vasoconstriction and clot formation, acting as a protein reservoir. Next, the wound undergoes an inflammatory process, in which vasodilation increases vascular permeability to promote chemotaxis; consequently, neutrophils and macrophages migrate to the wound site, mediating and ending a debridement process and secreting cytokines and growth factors. The third stage, known as the proliferative stage, consists in epithelization, angiogenesis, granulation tissue formation and collagen deposition, mediated by endothelial cells and fibroblasts. The fourth and last stage is remodeling, with collagen depositing in an organized manner, increasing the tensile strength of the wound. This last stage may last up to one year after wound healing has started [6,7]. In exceptional cases, where the injured tissues are unable to undergo complete regeneration, fibrous tissue will be deposited, creating scars [8].

Burns are one of the most common skin injuries and are the fourth most common type of trauma worldwide [9,10]. Regarding their severity, they can be classified and divided into three degrees, depending on the affected structures and area burned [3,11,12]. The classification of skin burns largely depends on which skin layers have been affected: in first degree burns, only the epidermis is affected; second degree burns usually extend to the dermal layer of the skin; while third degree burns implicate the hypodermis, sometimes completely burning through all the skin layers [13]. Whenever the skin is burned, its barrier function is reduced, making the body much more susceptible to infection [14]. In addition, extensive lesions that progress to adjacent tissue can lead to immunosuppression [12,14]. Moreover, burns can also lead to death by shock or septicemia caused by skin infection [15].

The treatment for burnt skin is described by many guidelines worldwide [16,17]. In general, these guidelines suggest the application of antimicrobial products, such as a silver sulfadiazine cream, followed by the application of a hydrocolloid to promote healing of the affected skin [18,19]. This therapeutic strategy, starting with an antibiotic and followed by a healing agent, is based on the premise that a non-infected burn heals faster.

Furthermore, innovative hydrocolloid dressings impregnated with silver also appear to be effective, increasing the interest in these devices [18]. However, the treatment of an infected burn is more difficult, prolonged and sometimes ineffective [20]. Although healing agents may promote skin regeneration, they simultaneously create a favorable environment for bacterial proliferation, which considerably delays healing time [20,21]. Thus, it is important to first apply an antibiotic or proceed with the concomitant application of an antibiotic agent with regenerating properties for these burns. Moreover, wounds can also be treated using dressings such as silver-based dressings that can release silver ions into the wound, simultaneously presenting antibacterial potential and promoting skin regeneration [22,23]. Currently, silver in topical sulfadiazine-based form is one of the marketed silver formulations for treating burns in the western half of the globe [24].

Nanotechnology emerged in the last century and its application in medicine, referred to as nanomedicine, has emerged mainly in the last three decades [25,26]. Silver nanoparticles (AgNPs) are widely used in several fields [25,27,28,29,30,31,32], for example, in the treatment of water, textile products, biosensors and storage of food products [33]. In healthcare, AgNPs are essentially used as an antimicrobial agent, but other metallic nanoparticles also present these properties (i.e., gold nanoparticles) [22,25,27,28,29,34,35]. In addition to antibacterial properties, AgNPs also present antiviral, antifungal, anti-inflammatory, anti-angiogenic, anti-tumoral and antioxidant activity, with applications as a delivery system in imageology and in cosmetics [33,34]. The antimicrobial activity of AgNPs relies on their physicochemical characteristics, such as size, shape, distribution and concentration [35,36,37,38,39]. Due to their high surface to volume ratio, AgNPs will require lower concentration and, consequently, leading to lower toxicity when compared with conventional silver, i.e., silver sulfadiazine or silver nitrate [36,37,38,39]. However, it is required that the formulation is maintained in the burn area. With this aim, a thermoreversible hydrogel was prepared.

Gels have a great importance for many applications [40]. The production of polymer-based gels started in the ‘70s, but in recent years, the interest in the physical gelation of polymers has increased [40,41]. This rise in interest is also explained due to hazard and toxic concerns related to conventional vehicles. In situ thermoreversible gels like hydrogels are alternative vehicle systems with many advantages [42]. Hydrogels are gels in which the dispersed phase is composed by water and the gelling agents are polymers [43]. They are three-dimensional (3D) hydrophilic polymeric networks able to retain large amounts of water or biological fluids and characterized by soft and rubbery consistence in analogy to living tissues. These gels are promising biomaterials due to their interesting properties such as biocompatibility, biodegradability, hydrophilicity and lack of toxicity [44]. These and other properties make hydrogels vehicles for many applications in the medical and pharmaceutical fields [44]. Pluronic^®^ F127 is an amphiphilic block copolymer, a poly(ethylene oxide)/poly(propylene oxide)/poly(ethylene oxide) (PEO–PPO–PEO) triblock copolymers, extensively explored for therapeutic applications mainly due to its appealing properties, such as being non-toxic, bioadhesive and stable, and presenting the ability to transform into a gel at physiologic temperature [45,46,47]. At low temperatures, Pluronic^®^ F127 is a solution but, as temperature increases, the hydrogen bonds in the hydrophilic chains of the Pluronic^®^ F127 copolymer desolvate, favoring hydrophobic interactions between the polyoxypropylene domains, forming a stable gel [46]. Due to its thermoreversible nature, Pluronic^®^ F127 becomes a gel upon administration, forming a physical and protective barrier [46,47].

This work aims to develop a semi-solid formulation of AgNPs, incorporated in a Pluronic^®^ F127 hydrogel, for topical application with antimicrobial and skin regeneration properties using three different methodological approaches. Compared to a commercial formulation based on silver ions, it is expected to achieve a comparable therapeutic effect. Several techniques and methodologies were applied to evaluate the skin permeation, antibacterial activity, in vitro and in vivo efficiency and safety properties of this new formulation.

## 2. Results

### 2.1. Physicochemical Characterization

**Figure 1:**
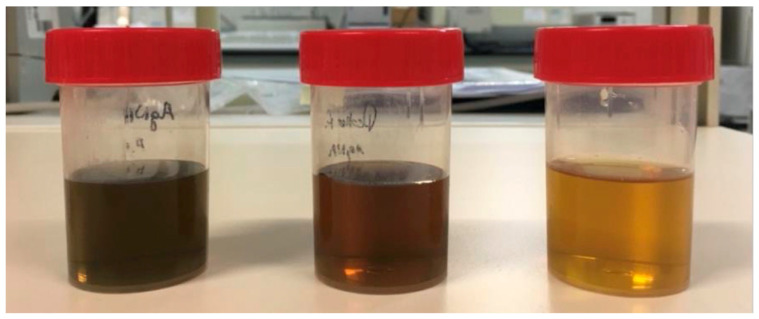
Macroscopic appearance of samples prepared by methods A, B and C (from **left** to **right**).

AgNPs were prepared following three different preparation methods, using the same reagents but differing in the temperature of the reagents used. The three methods (A, B and C) resulted in the successful preparation of AgNPs, but especially the described method C. As presented in Figure 1, the macroscopic appearance of the AgNPs prepared by this method resulted in a yellow translucid dispersion, in contrast to the AgNPs produced by methods A and B. Spectrophotometry analysis of AgNPs prepared by method C showed a characteristic absorption peak at 407 nm.

Table 1 presents results regarding mean size, polydispersity index (PdI) and zeta potential of AgNPs prepared using the different methods (A, B and C). Regarding particle size, the AgNPs prepared following method C presented a similar average size compared to AgNPs prepared following method A. However, the size dispersion of the AgNPs prepared by method C was lower than that of A and B, exhibiting lower PdI values, representing a more monodisperse formulation. Regarding its surface charge, determined by measuring the zeta potential, all of the prepared AgNPs have negative values of surface charge, close to neutrality (−10 to 10 mV), regardless of the preparation method employed, suggesting a potential biocompatibility.

**Figure 2:**
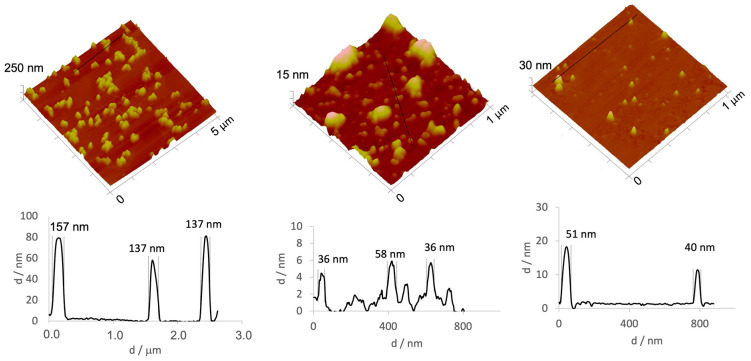
3D atomic force microscopy (AFM) images and corresponding profiles for AgNPs prepared following (**left**) method A, (**middle**) method B and (**right**) method C.

The morphology of the different AgNPs was assessed by AFM (Figure 2). Macroscopically, AgNPs suspension prepared by methods A, B or C were visually different, as previously mentioned, and the observed color changes are closely related to the presence of AgNPs’ aggregates. By analyzing the images obtained by AFM (Figure 2), it is possible to conclude that the AgNPs prepared by method A, presenting a greyish tone, contained clusters of much greater dimensions than that expected for free AgNPs, this being in accordance with data obtained by DLS (Table 1). The AFM images (Figure 2) show the presence of these aggregates for the AgNPs synthetized by methods A and B, corroborating the large PdI observed by DLS. In general, AgNPs present a non-spherical shape and sizes lower than 50 nm. Especially for AgNPs prepared by method A, large aggregates of AgNPs were consistently present, with dimensions between 100-200 nm. Taking into account these results, method C was selected for further tests as it yielded more homogeneous AgNPs, with a PdI below 0.2, when compared with the AgNPs prepared following methods A and B.

**Figure 3:**
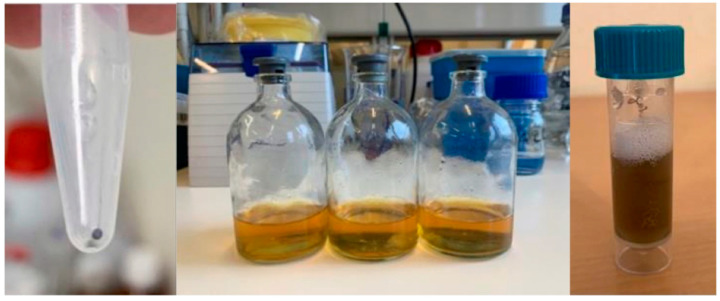
Pellet of the centrifuged sample (**left**), samples prepared for lyophilization (**middle**) and sample after solvent elimination by evaporation (**right**).

With method C selected, different recovery processes were also assessed to select the most suitable: centrifugation, lyophilization or solvent evaporation. The macroscopic results of recovering AgNPs using the mentioned processes are shown in Figure 3. AgNPs centrifugation led to the formation of a very dense pellet, impossible to resuspend, leading to the rejection of this recovery method. The same was observed for lyophilization, as the AgNPs were very difficult to resuspend in water. As for the vacuum rotary evaporator recovery method, it was much faster than lyophilization, and the AgNPs were easy to resuspend.

**Figure 4:**
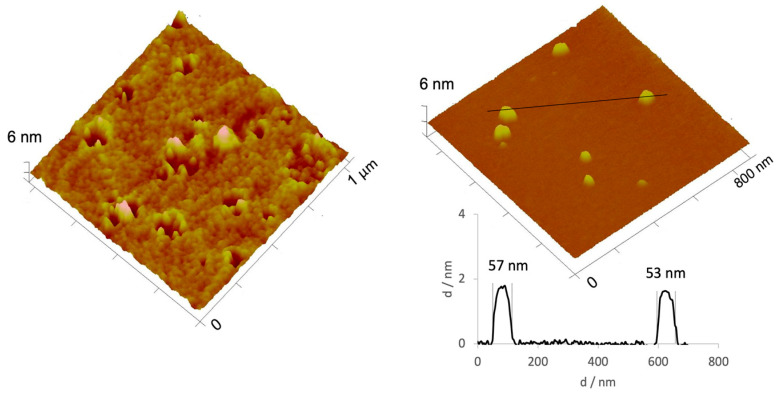
3D atomic force microscopy (AFM) images of non-diluted (**left**) and diluted (**right**) AgNPs recovered by solvent evaporation. A profile was added for the diluted sample.

To assess if recovering AgNPs by solvent evaporation using a vacuum rotary evaporator influenced AgNPs morphology, AFM images of AgNPs following solvent evaporation were obtained, for the non-diluted and diluted AgNPs (Figure 4). A very dense sample with considerable aggregates was observed in the non-diluted AgNPs recovered by a vacuum rotary evaporator. However, when the sample was diluted, it was very similar to the sample prepared by method C (Figure 4). This fact suggests that temperature has greater influence during the AgNPs formation (i.e., reagents temperature), but does not seem to have influence after AgNPs formation (i.e., AgNPs recovery). Thus, solvent evaporation using a vacuum rotary evaporator was the selected recovery method.

### 2.2. Antimicrobial Preliminary Efficacy Assessment

The bacterial strains used in this study were selected according to their tendency to colonize and infect burnt skin (i.e., *Escherichia coli*, *Staphylococcus aureus* and *Pseudomonas aeruginosa*) [48]. Table 2 displays the main results. Minimum inhibitory concentrations (MICs) were determined by broth microdilution for the three bacteria under study and for the different samples. The colloidal dispersion of AgNPs under the conditions of synthesis showed very little or no inhibition for most of the strains. In turn, when concentrated, whether by lyophilization, centrifugation or by evaporation, the bacterial inhibition was more expressive. Though there is inhibitory activity, MIC values are beyond the range of concentrations tested. However, AgNPs concentrated by lyophilization presented a more accentuated inhibitory activity against *P. aeruginosa*, and AgNPs concentrated by evaporation presented a more pronounced inhibitory activity against *E. coli* and *P. aeruginosa*, thus presenting higher efficacy against these strains.

### 2.3. In Vitro Permeation Studies

An in vitro skin permeation study was conducted for 24 h using an artificial membrane that mimics the skin. After 24 h, the amount of AgNPs that permeated the membrane was below the limit of detection of the analytical method used. This non-permeation of the AgNPs through the deep layers of the skin can be considered a good safety indicator.

### 2.4. Physical Characterization of Pluronic^®^ F127 Hydrogel

**Figure 5:**
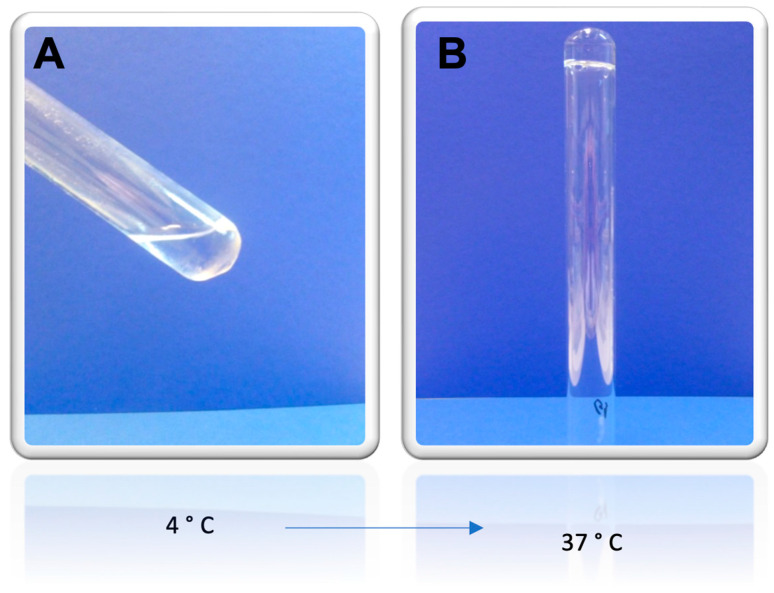
Macroscopic appearance of the thermoreversible Pluronic^®^ F127 hydrogel at (**A**) 4 °C, in liquid state, and (**B**) 37 °C, gelled.

In order for the AgNPs to remain in the burned region of the skin, a thermoreversible Pluronic^®^ F127 hydrogel was prepared and characterized regarding its viscosity and textural properties. Pluronics or Poloxamers are non-toxic FDA-approved poly(ethylene oxide)/poly(propylene oxide)/poly(ethylene oxide) (PEO-PPO-PEO) triblock copolymers. A variety of Pluronics is available on the market, differing for the molecular weight of the building blocks and the ratio between hydrophobic and hydrophilic units. Pictures of the prepared gel were taken at two different temperatures, in order to macroscopically characterize the gel. The obtained images are shown in Figure 5. At 4 °C (storage temperature), the hydrogel is in a liquid form, as can be seen in Figure 5A, and at 37 °C, physiologic temperature and the temperature at which the hydrogel will be applied, Pluronic^®^ F127 is in its gelled form, as shown in Figure 5B.

Viscosity is also a very important parameter for any semi-solid formulation. The viscosity was determined at different temperatures and results are shown in Table 3. Looking at the results, the thermoreversible properties of the Pluronic^®^ F127 hydrogel are apparent, as at 4 °C, the gel presented a viscosity of 75.7 ± 0.5 mPas, in its liquid form, and at 37 °C, a notorious higher viscosity, of 7333.3 ± 23.1 mPas.

The textural properties of the gel were also evaluated in triplicates, and the maximum peak force of displacement (F_max_), also denoted hardness, obtained was 0.6 ± 0.01 N. Viscosity and textural properties were also evaluated after the incorporation of the lyophilized AgNPs powder in the Pluronic^®^ F127 hydrogel, and its properties remain stable when compared to the hydrogel without nanoparticles.

### 2.5. In Vivo Efficacy and Safety Assessments

Skin burns started to appear on the second day of SDS application, and all animals completed the assay. Moreover, the animals did not present signs of stress or pain during the duration of the preliminary assessments.

**Figure 6:**
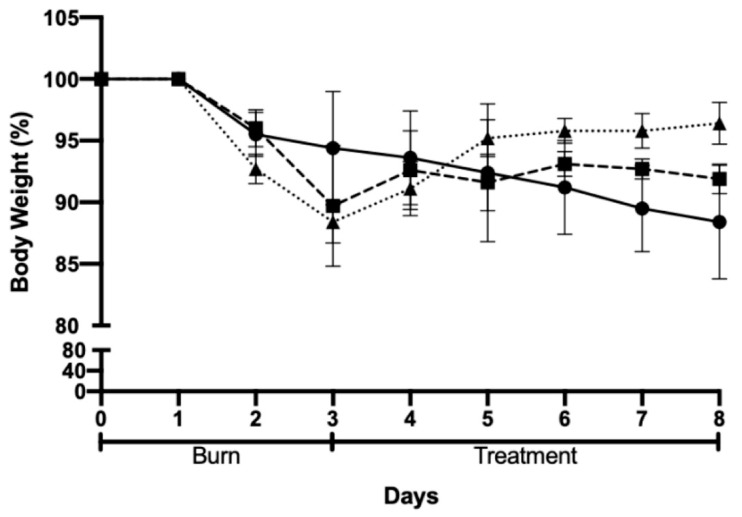
Body weight variation after chemically induced skin burn and treatment: Pluronic^®^ F127 hydrogel (negative control, circles); Positive control (commercial formulation of silver sulfadiazine squares); and test group, AgNPs incorporated in Pluronic^®^ F127 hydrogel (triangles).

The body weight of the animals was recorded for all groups, and the results are shown in Figure 6. The body weight of the animals decreased for all the groups following the burn induction but recovered after the beginning of treatment. Moreover, the body weight of all groups followed the same trend except for the negative control (Pluronic^®^ F127 hydrogel), in which body weight presented a smaller decrease following the skin burn when compared to the other groups. Furthermore, when comparing the body weights of the different groups on the last day of the assay, the group treated with AgNPs incorporated in Pluronic^®^ F127 hydrogel presented the best results, as the animals in this group recovered 96% of their initial body weight.

**Figure 7:**
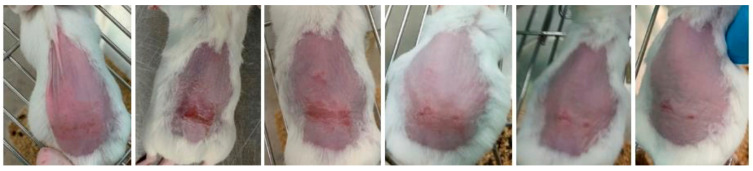
Progress of skin healing in test group treated with AgNPs incorporated in Pluronic^®^ F127 hydrogel (66 nM, from day 3 to day 8, following the course of treatment).

**Figure 8:**
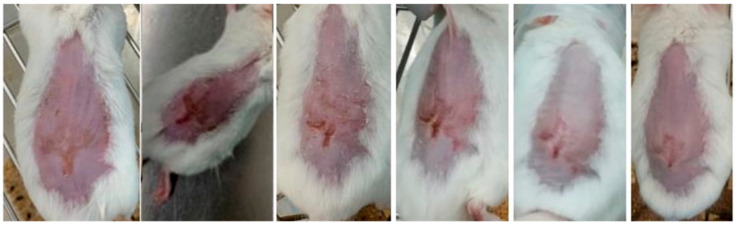
Progress of skin healing in the group treated with Pluronic^®^ F127 hydrogel (from day 3 to day 8, following the course of treatment).

**Figure 9:**
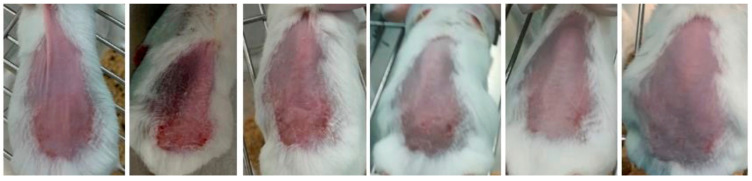
Progress of skin healing in the group treated with the positive control (commercial cream of silver sulfadiazine, 10 mg/g, from day 3 to day 8, following the course of treatment).

Representative images of mice from each group are shown in Figure 7, Figure 8 and Figure 9 and were taken daily after the beginning of the treatment. By evaluating the photographic records, it is possible to note that the AgNPs incorporated in Pluronic^®^ F127 hydrogel (Figure 7) caused rapid skin regeneration in the test group, with the skin practically healthy at the end of the treatment schedule and without noticeable scarring. In contrast, the negative control (Figure 8), Pluronic^®^ F127 hydrogel, led to a pronounced scar. Regarding the positive control (Figure 9), the commercial topical formulation of silver sulfadiazine led to a complete regeneration of the skin at the same time as the test group, without leaving any noticeable scars on the skin. However, the concentration of silver in the positive control (15.3 μmol of silver per cm^2^) was not equivalent, i.e., AgNPs were administered at a very low dose (3.3 pmol of silver per cm^2^).

**Figure 10:**
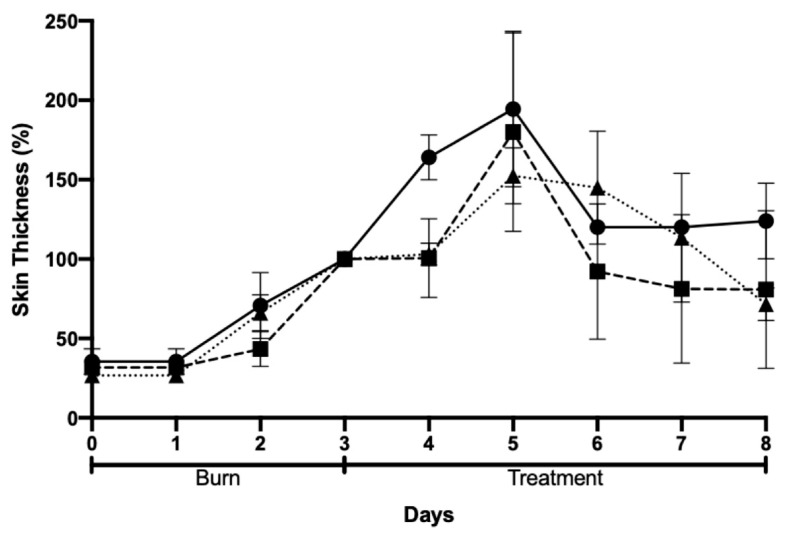
Skin thickness after chemically induced burn and treatment: negative control (Pluronic^®^ F127 hydrogel, circles); positive control (commercial cream of silver sulfadiazine, squares); and AgNPs incorporated in Pluronic^®^ F127 hydrogel (triangles).

Skin thickness was also evaluated and tended to increase with the progression of the injury due to inflammation and subsequent skin regeneration with the formation of crusts. This increase was quite consistent across the different groups (Figure 10). An ideal result would be the achievement of a similar skin thickness the burn and at the end of the protocol. This did not happen for any of the groups under study. However, results showed a higher tendency of improvement in skin thickness for animals treated with AgNPs incorporated in Pluronic^®^ F127 hydrogel.

### 2.6. Histopathological Analysis

**Figure 11:**
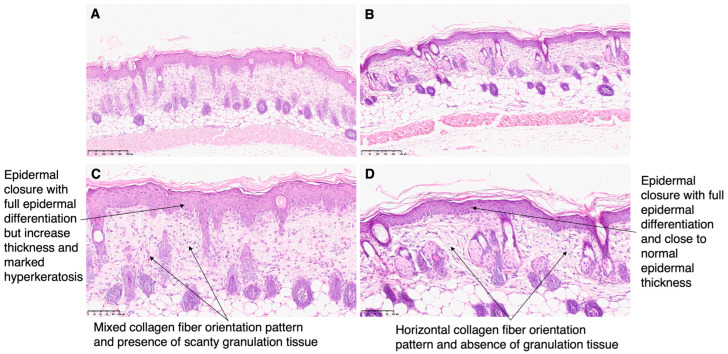
Histological section images of the skin of the mice after 5 days of treatment with (**A**,**C**) commercial cream of silver sulfadiazine (10 mg/g), and (**B**,**D**) AgNPs incorporated in Pluronic^®^ F127 hydrogel (66 nM). (**A**,**B**) Magnification 10× and (**C**,**D**) magnification 20×.

The burns were histologically analyzed and representative images are presented in Figure 11. Both skin of mice treated with silver sulfadiazine and AgNPs incorporated in Pluronic^®^ F127 presented epidermal closure with full epidermal differentiation, but skin of mice treated with the positive control presented increased thickness and marked hyperkeratosis. Moreover, the skin of mice in the positive control group presented scanty granulation tissue and mixed orientation of the collagen fibers, while granulation tissue was absent in the test group, and the collagen was present in a horizontal pattern. Moreover, a previously described skin regeneration scoring system was used to compare the burns of all animals that participated in this preliminary study (Table 4). The chosen scoring system takes different aspects into account such as the presence of an ulcer or if the wound is completely closed, the degree of epidermis differentiation, the amount of granulation tissue present, and lastly, the collagen fibber orientation and pattern. A score of 16 indicates full regeneration of the skin lesion while lower scores refer to the presence of histologic changes compatible with skin injury. The highest score was of animals treated with AgNPs incorporated in Pluronic^®^ F127 hydrogel (test group, score of 14.60 ± 3.13). It is to be noted that the lowest score was seen in the animals of the positive control group, treated with a commercial formulation of silver sulfadiazine. This animal model was previously validated by our group, and the same scoring system was used to score the untreated burns of this animal model, obtaining a score of 12.0 ± 2.8 [9]. Thus, the skin of mice treated with AgNPs incorporated in Pluronic^®^ F127 presented an advanced wound healing.

In the set of seven mice, only two of them did not have a maximum score indicating why the skin was not fully regenerated. One of these animals was allocated to the group treated with the commercial formulation of silver sulfadiazine. The skin was ulcerated and showed no differentiation at the level of the epithelium. The granulation tissue and the inflammatory infiltrate had moderate levels. Collagen appeared in a vertical orientation with a reticular pattern. The total score for this mouse in the group treated with the commercial formulation of silver sulfadiazine was 6. The other mice that did not reach the maximum score were part of the group treated with AgNPs incorporated in Pluronic^®^ F127 hydrogel. In this case, contrary to the situation described above, the lesion was already closed and the collagen was already in a more approximate to normal disposition. However, both inflammatory infiltrate and granular tissue are present, indicating that skin regeneration would not be completed. Thus, the final score for this animal was 9.

Although the skin of most animals, independently of treatment or control groups, had completely recovered, as corroborated by the scoring system, the macroscopic analysis allowed for the classification of animals into two distinct categories: recovered with scarring and recovered without scarring. As seen in Figure 8, the animal representative of the negative control group had regenerated skin but presented a pronounced scar, unlike what happened with the animals of the other groups (Figure 11). The spleen was also subjected to analysis to check for possible toxicity, and all animals in the study showed no changes in this organ.

## 3. Discussion

Different methods have been described to prepare AgNPs, and these can be of three types: physical (i.e., evaporation–condensation, laser ablation), chemical (i.e., reduction, electrochemical and photochemical methods) or biological (i.e., based on oxidation-reduction reactions mediated by microorganisms such as bacteria, fungi or plant extracts) [49,50,51,52]. Amongst these methods, the most commonly used is chemical reduction, as it has a low cost of production, high performance and is fairly simple [49,52]. As described above, this method uses a solution of NaBH_4_ to reduce Ag^+^ to Ag^0^, forming a cluster and originating colloidal AgNPs [49,50,52]. To obtain a monodisperse AgNPs, all nuclei must be formed at the same time, to consequently have the same growth, and this is dependent on pH and temperature [53]. In this work, it is worth noting that preparation methods A, B and C differed in the temperature of the reagents, and it seems that the temperature of the reagents used in the preparation of the AgNPs influences their size, dispersion, morphology and the presence of aggregates. On the other hand, AgNPs suspension produced by method C showed a yellow color that previous studies claim to be indicative of the formation of AgNPs without aggregates. In a previous study, AgNPs produced at 50 °C presented a brown color after synthesis. When analyzed by AFM, the produced AgNPs were large (~50 nm), non-spherical and presented several aggregates. When the same preparation method was used but using cooled reagents, at a temperature of 10 °C, the reaction speed was slower and the initial yellow color, seen right after synthesis, only shifted after a few hours [53].

The influence of the particle size on the antimicrobial activity of AgNPs is not consensual [33,54,55,56]. Although different bacterial species differ in the size ranges of AgNPs that inhibit their bacterial growth, most bacterial growth is inhibited by smaller AgNPs (<50 nm) [57,58]. Martínez-Castañón et al. compared the MIC’s of AgNPs with different sizes (7, 29 and 89 nm), demonstrating that smaller AgNPs (7 nm) presented higher antibacterial activity against *E. coli* and *S. aureus* [59]. In another study, Jeong et al. also compared the antimicrobial activity of AgNPs with 10 and 100 nm. AgNPs with the size of 10 nm showed comparable antibacterial activity against *Methylobacterium* spp. with the positive control (methanol) [60]. Skin penetration rates and depth-of-penetration play significant roles in determining the therapeutic potential of topical agents and their systemic toxicity. Theoretically, the smaller the size of the particles, the higher the rate of penetration. In our case, AgNPs produced by method C presented greater homogeneity (PdI of 0.180). Although NPs are mostly preferred for their large surface area, smallness should not be a core goal, as the physicochemical properties of NPs can be efficiently utilized in topical antimicrobial formulations. A study carried out with gold NPs showed that smaller particles with 15 nm reached the deepest layers of the mouse skin while NPs with sizes of 102 and 198 nm only reached the epidermis and the dermis [61]. Another study with polymeric NPs demonstrated that particles with a diameter of approximately 300 nm did not permeate the human skin during the 6 h after their application without mechanical stress (passive permeation) [62]. This was shown by Ezealisiji et al., who tested the skin penetration of AgNPs with 22, 58, 76 and 378 nm [63]. Thus, considering the size of the herein developed AgNPs formulation following method C (ca. 50 nm), our AgNPs should only reach the most external layers of the skin, aiming at the goal of this study and potentially decreasing the probability of the systemic absorption.

Silver itself is non-toxic to humans within the reference dose, i.e., oral reference dose (RfD) = 5× 10^−3^ mg/kg-day [64]. Overconsumption of silver, however, may lead to argyria, which results in permanent blue-grayish pigmentation of the skin, eyes and mucous membranes. Systemic toxicity can be caused by rapid accumulation of NPs at capillary/lymphatic junctions in the dermal layer, membrane pores/ligand-mediated endocytosis and physically breached leaky endothelium. Rapidly penetrating NPs could circumvent macrophage-mediated immunological responses and can enter the blood circulatory system. In contrast, slow penetration offers better efficacy of NPs against the infected cells and provides adequate time for the body’s immune system to detoxify NPs through phagocytosis. In this work, the skin permeation study did not detect any measurable value of silver, which is a good indicator regarding putative systemic toxic effects.

Besides the particle size, surface charge, determined by zeta potential, is a measure of stability of colloidal dispersion, for which higher values in the module indicate greater physical stability of the dispersion under analysis and less tendency to form aggregates. It is described how negatively charged AgNPs diffuse through the skin at a greater speed [65]. In turn, particles that have a positive zeta potential (above 10 mV) are more likely to bind cells and be recognized by the immune cells [29]. Therefore, particles developed in this work presented a surface charge close to neutrality (between −10 and 10 mV) and would be ideal due to presenting low or no skin penetration and increased biocompatibility. Martínez-Higuera et al. developed negatively charged AgNPs, incorporated in a Carbopol^®^ hydrogel with *Mimosa tenuiflora* extracts, demonstrating the wound healing potential of AgNPs [66].

The condition of the skin (i.e., whether intact or damaged) is another factor that influences the ability of AgNPs to penetrate the skin. A study conducted by Larese et al. showed that AgNPs with a mean size of 25 nm had an increase in skin penetration when the skin was damaged (2.32 ng/cm^2^) when compared to intact skin (0.46 ng/cm^2^), in vitro [67,68]. When the AgNPs are able to penetrate the skin, several works have shown that these particles usually precipitate at the stratum corneum, preventing AgNPs from precipitating into deeper layers of the skin [68,69,70]. Contrary to these studies, an in vivo study conducted by George et al. on normal intact skin showed that AgNPs penetrate into deeper layers of the skin, the reticular dermis, and thus, AgNPs are not retained in the stratum corneum. Regardless, none of these studies reported the presence of AgNPs in systemic circulation [68].

In the present study, it was also observed that the therapeutic effect of the resultant AgNPs prepared by method C varied for different tested bacteria, probably due to the disparity in the way AgNPs interact with different bacterial strains [48,71,72,73]. When compared to the commercialized formulation, the developed AgNPs outperformed the commercial silver sulfadiazine regarding in vitro antimicrobial activity for the tested strains, using lower concentrations of silver.

The exact mechanism of action is not completely understood. After contacting with the skin, one of the main concerns with AgNPs is the possible depletion of mitochondrial function with the production of ROS. Results from an in vitro study carried out on 3D-fibroblast cultures demonstrated that the reduction in mitochondrial activity only occurred temporarily and did not affect their viability [74]. In addition, an in vivo study of a biopsy of AgNPs-treated skin of a single patient showed a large amount of AgNPs without showing signs of apoptosis or necrosis, which corroborates the absence of toxicity [74]. From the results of these studies, it can be concluded that the toxicity conferred by silver is not as accentuated as initially thought. Regarding the possible systemic toxicity of AgNPs, the skin permeation study did not show any silver over time.

Semi-solid formulations have been indicated for better consumer acceptance of the treatment and allow for good skin-spread ability of the formulation [75]. In particular, hydrogels leave a semi-transparent layer over the burn, allowing a burn protection from the external environment and accelerate the wound healing processes [43]. Thus, in order to increase the contact time of AgNPs with the burn site and promote the wound healing properties of the AgNPs, the lyophilized AgNPs were incorporated in a semi-solid formulation, a Pluronic^®^ F127 hydrogel. AgNPs synthesized by other groups have been incorporated into hydrogels based on other polymers, e.g., Masood et al. impregnated a chitosan-PEG hydrogel with AgNPs [76], Nguyen et al. loaded AgNPs into chitosan/Polyvinyl Alcohol hydrogel [77], Ahsan et al. used a PVA hydrogel for AgNPs-hydrogel patches [78], Xie et al. reinforced chitosan hydrogels with AgNPs [79] and Badhwar et al. loaded quercetin hydrogels with AgNPs [80], with wound-healing applications. In fact, Pluronic^®^ F127 presents unique features, such as being thermoreversible, even at low concentrations, being liquid at temperatures lower than the physiological temperature, at which it becomes a gel [47,81,82,83]. This has led Pluronic^®^ F127 to be vastly researched for dermal and transdermal applications [46,83].

The wound-healing process was treatment dependent. The test group, treated with AgNPs incorporated in Pluronic^®^ F127 hydrogel, had a skin regeneration score below 16. Although this indicates that the wound of the animals in the test group is yet to completely heal, it was still higher than the skin regeneration score of the animals in the positive control group, treated with a commercialized silver sulfadiazine cream. As the AgNPs incorporated in Pluronic^®^ F127 hydrogel presented a much lower concentration of silver in comparison to the commercialized silver sulfadiazine cream, this result is very promising, as the AgNPs were more effective in treating the skin burns in this chemically induced burn in vivo mice model than the positive control. Zhang et al. analyzed the delayed treatment of burns with AgNPs and besides results being also very promising, the wound healing was slower and a higher concentration of silver was used [84]. Comparing our in vivo results with the work developed by Stojkovska and colleagues, this was also the case, in which burns were treated with formulations containing alginate and AgNPs but, again, with a higher concentration of silver [85]. Posteriorly, biochemical analysis, to quantify inflammatory and pro-inflammatory factors in the animals’ serum, and identification of the bacterial species colonizing the scarred burn were also performed. Biochemical analysis demonstrated that the animals in the test group did not present an inflammatory response, as the values for IL-6 and TNF-α were 0 pg/mL and <4 pg/mL, respectively. Moreover, the bacterial strains identified at the scarred burn site were common between the different experimental group and were consistent with environment and/or fecal contaminations and/or colonization (i.e., *S. aureus*, *Enterococcus faecalis*, *Staphylococcus xylosus* and *Micrococcus luteus*), as these strains are commonly found in the environment, feces and/or commensal bacteria of the skin, indication that the burns did not become infected. We hope that our proof-of-concept study could facilitate a new paradigm for understanding NPs while developing an ideal antimicrobial topical formulation.

## 4. Conclusions

The current study explores the application of silver nanoparticles (AgNPs) as a topical treatment of skin burns, one of the most common types of skin injury in the world, with antibacterial and wound-healing properties. In addition, this work explores the use of a thermoreversible hydrogel to deliver those AgNPs. 

Skin therapy using hydrogel drug delivery systems has been gaining attention because of its dual functionality to simultaneously supply moisture and loaded actives onto infected sites on the skin. AgNPs were successfully prepared by a chemical reduction method with sodium borohydride at a specific temperature. Different methods were assessed, differing in reagents’ temperature only. According to the results obtained in the various assessments, it was observed that temperature differences when producing AgNPs have a significant impact on its physicochemical characteristics. Moreover, the method that yielded AgNPs with more desirable physicochemical characteristics used sodium borohydride at a lower temperature, after being stored at −18 °C for 10 min, allowing the reaction to occur at a slower speed. Of the different recovery processes assessed, solvent elimination by evaporation resulted in AgNPs with efficient bacterial growth inhibition that were easily dispersed in water and maintained AgNPs properties.

In vivo studies using a chemical burn model showed that AgNPs incorporated in Pluronic^®^ F127 hydrogel, at a lower concentration of silver, performed similarly to the positive control, a commercialized formulation of silver sulfadiazine with higher silver concentration, in terms of skin thickness and wound healing. The water-rich structure of the hydrogel and wound-healing properties of AgNPs seem to have the required dual-characteristics for the treatment of skin burns, since they have high efficacy when topically used, since it requires smaller concentrations of silver for the treatment of burns compared to formulations found in the market and skin regeneration was effective using new safe and hydrogel-based materials.

## 5. Materials and Methods

### 5.1. Materials

#### 5.1.1. Reagents

Silver nitrate (AgNO_3_, Cat. No. 209139), Pluronic^®^ F127 (Cat. No. P2443) and sodium borohydride (NaBH_4_, Cat. No. 71320) were purchased from Sigma Aldrich (Steinheim, Germany). Milli-Q water was obtained in filtration equipment from Millipore Corporation (Burlington, MA, USA). A commercial silver-based formulation in which each gram of cream contains 1 mg of micronized silver sulfadiazine was purchased. Reaction buffer for NZY TaqII DNA polymerase (Cat. No. MB354), NZYTaq II DNA polymerase (Cat. No. MB355) 5 U/μL, magnesium chloride 50 mM, a standard solution, NZYDNA Ladder VI (Cat. No. MB089), agarose powder of routine grade, GreenSafe Premium dye (Cat. No. MB13201) and NZYGelpure (Cat. No. MB011) kit were purchased from Nzytech (Lisbon, Portugal). All other reagents were of analytical grade.

#### 5.1.2. Microbial Strains

The in vitro antimicrobial study was carried out using Gram-positive bacteria (*Staphylococcus aureus*, ATCC 29213) and Gram-negative bacteria (*Escherichia coli*, ATCC 25922 and ATCC 8739, and *Pseudomonas aeruginosa*, ATCC 27853).

#### 5.1.3. Animals

Eight-week-old female CD-1 mice (25–40 g), obtained from Charles River (Barcelona, Spain) were housed in polypropylene cages in a 12–12 h light-dark cycle with a constant temperature environment of 20–24 °C, relative humidity of 55 ± 5% and received standard diet and water *ad libitum*. All animal experiments were conducted according to the recommendations of the Animal Welfare Board (ORBEA) of the Faculty of Pharmacy, *Universidade de Lisboa*, approved by the competent national authority Direção-Geral de Alimentação e Veterinária (DGAV) for project with reference PTDC/BBBBMD/0611/2012, DGAV/2013, and per the EU Directive (2010/63/EU), the Portuguese laws (DL 113/2013, 2880/2015, 260/2016 and 1/2019), and all relevant legislation.

### 5.2. Methods

#### 5.2.1. Preparation of AgNPs

A solution of AgNO_3_ (1 mM) was added dropwise to a NaBH4 (2 mM) solution under constant stirring. The optimization of the synthesis protocol was achieved by varying the temperature of the solutions used for preparation (Table 5) and thus, different batches of AgNPs were prepared (method A, B and C).

The AgNPs suspension was protected from light with aluminum foil, at 4 °C. Synthesis of AgNPs was confirmed by spectrophotometry (UV-Vis Spectrophotometer, Hitachi U-2000 Dual-Beam UV-Vis, Oxford, United Kingdom) in which an absorption peak around 400 nm should be present.

Recovery of the AgNPs was performed by three different methods. The first one consists of centrifuging the prepared AgNPs (Sigma 3-30KS, Sigma Zentrifugen, Osterode am Harz, Germany) at 60,000× *g* for 20 min, followed by 40 min at 40,000× *g* at 4 °C; the solvent elimination by rotary vacuum evaporator method was adapted from a previous study in which a vacuum rotary evaporator (Butchi RE 111, Butchi, Switzerland) in a hot bath (70 °C, Butchi 461 Water Bath, Butchi, Switzerland) was used; lastly, lyophilization was carried out by leaving AgNPs in a freeze dryer (Modulyo, Edwards, CO, USA) at 102 mbar for 24 h.

#### 5.2.2. AgNPs Characterization

- Mean Size and Surface Charge

AgNPs diluted in Milli-Q water (1:10, pH 7) were analyzed in terms of size and polydispersity index (PdI) by Dynamic Laser Scattering (DLS) (Nano Z Zetasizer, Malvern Instruments, Malvern, United Kingdom).

The AgNPs’ surface charge was measured through Laser Doppler Anemometry (Nano Z Zetasizer, Malvern Instruments, Malvern, United Kingdom). For this measurement, samples were diluted in NaCl 0.1 M solution (1:10, pH 7).

- Morphology Analysis

The morphology of the nanoparticles was assessed by atomic force microscopy (AFM). Briefly, 40 µL of the sample was placed on a freshly cleaved mica surface. The mica was left to dry overnight and analysis was performed the following day. Images were acquired by Multimode 8 HR coupled to Nanoscope V (Bruker, Billerica, MA, USA) using Peak Force Tapping and ScanAssist AFM mode and silicon nitride ScanAsyst-Air probes (spring constant of ca.0.4 N/m, Bruker).

- Quantification of AgNPs

Finally, AgNPs were quantified using spectrophotometry. A calibration curve was prepared, and the following equation was obtained (R^2^ = 0.998):

(1) $$\mathrm{Abs}=0.198C+9.209\times 10^{-3}$$

where Abs is absorbance, and C is AgNPs concentration.

#### 5.2.3. Antimicrobial Preliminary Efficacy Studies

All strains used were kept stored at −80 °C and were previously grown overnight at 37 °C in Muller–Hinton Agar plates. All antimicrobial assays were carried out by broth microdilution in Muller–Hinton broth in non-treated 96-well plates containing two-fold dilutions of the compound/formulation tested. The inoculum was prepared by suspending overnight bacterial growth in sterile distilled water as to obtain a bacterial suspension adjusted to a 0.5 McFarland standard, followed by 1:100 dilution in Muller Hinton broth. An equal volume to that present in each well was used to inoculate the plates to obtain a final inoculum concentration of ca. 5.0 × 10^5^ colony-forming units (CFU)/mL. Compound-free and non-inoculated wells were included in each plate as positive and negative controls, respectively. The plates were incubated overnight at 37 °C and the Minimum Inhibitory Concentration (MIC) determined as the lowest concentration that inhibited visual growth by each strain.

#### 5.2.4. In Vitro Skin Permeation Studies

Permeation studies were conducted with Franz cells using a silicon membrane, in a water bath at 32 °C for 24 h. The donor chamber was filled with 300 μL of AgNPs (2 mg/mL) using Tween^®^80 (0.04%, *v*/*v*) as a dispersing agent, and the receptor chamber was filled with PBS at pH 7.4 (USP39) under constant stirring (100 rpm). Samples were collected every hour for the first eight hours, as well as all of the cell content at the end of the study being finished. The concentration of AgNPs was accessed by spectrophotometry, following the above-described method.

#### 5.2.5. Preparation and Physical Characterization of Pluronic^®^ F127 Hydrogel

For in vivo assessment of AgNPs and to deliver these nanoparticles to the target (burn) area, a thermoreversible hydrogel was prepared. Twenty-eight grams of Pluronic^®^ F127 were added to 100 mL of phosphate buffer solution (PBS pH 7.4, USP32) in a beaker, according to a previous study [86]. The solubilization was then carried out using a magnetic plate (100 rpm, 2 h). The prepared Pluronic^®^ F127 hydrogel was stored at 4 °C. Viscosity was determined using 100 rpm with needle n.º 3, in triplicate (*n* = 3), at different temperatures (4, 25 and 37 °C) using a Brookfield^®^ Rotational Viscometer (Middleborough, MA, USA). Texture analysis (firmness and adhesiveness) was performed using the Stable Micro Systems TA-XT2i Texturometer (Godalming, United Kingdom). A test probe P/25P (25 mm/s) was used, with a test speed of 3 mm/s, distance 5 mm, load cell with 5 k and Trigger Force of 0.049.

#### 5.2.6. In Vivo Efficacy and Safety Assays: Proof of Concept

Animals were randomly allocated into three experimental groups: a group dosed with the vehicle of the test formulation (*n* = 3); a group dosed with a commercial cream of silver sulfadiazine (10 mg/g of cream) (*n* = 3); a test group dosed with AgNPs (66 nM) dispersed in Pluronic^®^ F127 hydrogel (*n* = 5). Each application of commercial cream of silver sulfadiazine corresponds to 15.3 μmol of silver per cm^2^ and each application of AgNPs dispersed in Pluronic^®^ F127 hydrogel corresponds to 3.3 pmol of silver per cm^2^.

Before experimentation began, animals were lightly anaesthetized with isoflurane and an area of 2 cm^2^ of the back of each mouse was shaved with a commercial depilatory cream to expose the skin. Then, a chemical burn was induced by topical application of 100 μL Carbopol 940^®^ gel containing 40% of SDS for two consecutive days.

All formulations were administered topically (100 µL) with a syringe and performed daily, during 5 days of protocol, after light sedation with isoflurane. After administration, the water provided to the animals contained codeine (30 mg/500 mL) to reduce any pain and ensure welfare. During the 8-day experiment (since time zero), the body weight, skin thickness (Fisherbrand^TM^ Traceable^TM^ Carbon Fiber Calipers 6”, FisherScientific, Hampton, NH, USA) and welfare of all animal groups were monitored. The burns were photographed each day to record burn evolution. A sterile swab was used to collect the skin flora of animals in each group for bacterial strain identification.

After 8 days, the animals were sacrificed and approximately 1 cm^2^ of the burn area was harvested from each mouse, along with the spleen, and stored in formalin for histological analysis. Biochemical analysis was performed on the serum of all animals in each group to quantify IL-6 and TNF-α.

#### 5.2.7. Histology

Specimens of skin and spleen were excised and fixed in 10% buffered formalin for a minimum period of 48 h and were routinely processed, embedded and sectioned into 3 μm thick sections, and stained with H&E. Slides were analyzed with a CX31 microscope (Olympus Corporation, Tokyo, Japan), and images were acquired with the NanoZoomer-SQ Digital slide scanner C13140-01 (Hamamatsu Photonics, Shizuoka, Japan).

A scoring system for wound healing was developed by adaptation of previously published scoring systems [9,87]. Briefly, skins were scored for epidermal closure (0—ulcerated skin, 1—closed wound); epidermal differentiation (0—absent, 1—spinous epidermal, 2—granular layer); amount of granulation tissue (1—profound, 2—moderate, 3—scanty, 4—absent); inflammatory infiltrate (1—plenty, 2—moderate, 3—few); collagen fiber orientation (1—vertical, 2—mixed, 3—horizontal) and pattern of collagen (1—reticular, 2—mixed, 3—fascicle).

#### 5.2.8. Statistical Analysis

Results were expressed as mean ± standard deviation (SD) for in vitro studies. For biological assays, results were expressed as mean ± standard error of the mean (SEM).

## Figures and Tables

**Table 1:** Mean size, polydispersity index (PdI) and zeta potential (mean ± SD).

Preparation Method	Size (nm)	PdI	Zeta Potential (mV)
A	47.85 ± 1.01	0.887 ± 0.003	−0.06 ± 1.50
B	18.92 ± 2.61	0.628 ± 0.162	−2.02 ± 0.34
C	48.04 ± 14.87	0.180 ± 0.013	−0.79 ± 2.17

**Table 2:** Minimum inhibitory concentrations (MIC) for *Escherichia coli*, *Staphylococcus aureus* and *Pseudomonas aeruginosa* (*n* = 3, presented values are coincident between *n* ≥ 2).

Sample	MIC (nM)		
	*Escherichia coli*	*Staphylococcus aureus*	*Pseudomonas aeruginosa*
AgNPs	>1.65	>1.65	1.65
Centrifuged AgNPs (66 nM)	>33	>33	>33
Lyophilized AgNPs (66 nM) + Pluronic^®^ F127 hydrogel	>33	>33	33
AgNPs concentrated by evaporation (66 nM)	33	N/A	33

**Table 3:** Viscosity of the Pluronic^®^ F127 hydrogel at different temperatures.

Time (min)	Viscosity (mPas)		
	4 °C	25 °C	37 °C
10	76.0	360.0	7320.0
20	76.0	380.0	7360.0
30	75.2	360.0	7320.0
Mean ± SD	75.7 ± 0.5	366.7 ± 11.6	7333.3 ± 23.1

**Table 4:** Scoring of skin regeneration following 5 days of treatment, as follows: epidermal closure (0—ulcerated skin, 1—closed wound), epidermal differentiation (0—absent, 1—spinous epidermal, 2—granular layer), amount of granulation tissue (1—profound, 2—moderate, 3—scanty, 4—absent), presence of inflammatory infiltrates (1—plenty, 2—moderate, 3—few), the orientation of collagen fibers (1—vertical, 2—mixed, 3—horizontal) and collagen pattern (1—reticular, 2—mixed, 3—fascicle) [9,38].

	Epidermal Closure	Epidermal Differentiation	Amount of Granulation Tissue	Inflammatory Infiltrate	Orientation of Collagen Fibers	Collagen Pattern	Total
Commercial formulation of silver sulfadiazine (*n* = 3)	0.67 ± 0.58	1.33 ± 1.15	3.33 ± 1.15	2.67 ± 0.58	2.33 ± 1.15	2.33 ± 1.15	12.67 ± 5.77
AgNPs incorporated in Pluronic^®^ F127 hydrogel (*n* = 4)	1.0 ± 0.0	1.60 ± 0.89	3.33 ± 0.89	2.80 ± 0.45	2.80 ± 0.45	2.80 ± 0.45	14.60 ± 3.13

**Table 5:** Temperature of the solutions used for AgNPs production following methods A, B and C.

Method	Conditions
A	AgNO_3_ was cooled prior to the preparation of the AgNO_3_ solution, and the solutions used to prepare the AgNPs were at room temperature.
B	The AgNO_3_ and NaBH_4_ solutions were prepared and stored at 4 °C prior to AgNPs production.
C	AgNO_3_ solution was produced and used at room temperature, while NaBH_4_ solution was cooled prior to being used in AgNPs production.

## Data Availability

Not applicable.

## References

[1] Reis C.P., Gomes A., Rijo P., Candeias S., Pinto P., Baptista M., Martinho N., Ascensão L. (2013). Development and Evaluation of a Novel Topical Treatment for Acne with Azelaic Acid-Loaded Nanoparticles. Microsc. Microanal..

[2] Proksch E., Brandner J.M., Jensen J.-M. (2008). The skin: An indispensable barrier. Exp. Dermatol..

[3] Kalantari K., Mostafavi E., Afifi A.M., Izadiyan Z., Jahangirian H., Rafiee-Moghaddam R., Webster T.J. (2020). Wound dressings functionalized with silver nanoparticles: Promises and pitfalls. Nanoscale.

[4] Mota A.H., Rijo P., Molpeceres J., Reis C.P. (2017). Broad overview of engineering of functional nanosystems for skin delivery. Int. J. Pharm..

[5] Reis C.P., Damgé C. (2012). Nanotechnology as a Promising Strategy for Alternative Routes of Insulin Delivery. Methods Enzymol..

[6] Politano A.D., Campbell K.T., Rosenberger L.H., Sawyer R.G. (2013). Use of Silver in the Prevention and Treatment of Infections: Silver Review. Surg. Infect..

[7] Ovais M., Ahmad I., Khalil A.T., Mukherjee S., Javed R., Ayaz M., Raza A., Shinwari Z.K. (2018). Wound healing applications of biogenic colloidal silver and gold nanoparticles: Recent trends and future prospects. Appl. Microbiol. Biotechnol..

[8] Guillamat-Prats R. (2021). The Role of MSC in Wound Healing, Scarring and Regeneration. Cells.

[9] Quitério M., Simões S., Ascenso A., Carvalheiro M., Leandro A.P., Correia I., Viana A.S., Faísca P., Ascensão L., Molpeceres J. (2021). Development of a Topical Insulin Polymeric Nanoformulation for Skin Burn Regeneration: An Experimental Approach. Int. J. Mol. Sci..

[10] Greenhalgh D.G. (2019). Management of Burns. N. Engl. J. Med..

[11] Reinke J.M., Sorg H. (2012). Wound Repair and Regeneration. Eur. Surg. Res..

[12] Liu S.-H., Huang Y.-C., Chen L.Y., Yu S.-C., Yu H.-Y., Chuang S.-S. (2018). The skin microbiome of wound scars and unaffected skin in patients with moderate to severe burns in the subacute phase. Wound Repair Regen..

[13] Abraham J.P., Plourde B.D., Vallez L.J., Nelson-Cheeseman B.B., Stark J.R., Sparrow E.M., Gorman J.M. (2018). Skin Burns. Theory and Applications of Heat Transfer in Humans.

[14] Norman G., Christie J., Liu Z., Westby M.J., Jefferies J.M., Hudson T., Edwards J., Mohapatra D.P., Hassan I.A., Dumville J.C. (2017). Antiseptics for burns. Cochrane Database Syst. Rev..

[15] Zhang P., Zou B., Liou Y.-C., Huang C. (2021). The pathogenesis and diagnosis of sepsis post burn injury. Burn. Trauma.

[16] Yoshino Y., Ohtsuka M., Kawaguchi M., Sakai K., Hashimoto A., Hayashi M., Madokoro N., Asano Y., Abe M., Ishii T. (2016). The wound/burn guidelines—6: Guidelines for the management of burns. J. Dermatol..

[17] Ahuja R.B., Gibran N., Greenhalgh D., Jeng J., Mackie D., Moghazy A., Moiemen N., Palmieri T., Peck M., ISBI Practice Guidelines Committee (2016). ISBI Practice Guidelines for Burn Care. Burns.

[18] Wiktor A., Richards D., Torrey S.B. (2017). Treatment of Minor thermal Burns. https://www.medilib.ir/uptodate/show/349.

[19] Lloyd E.C.O., Rodgers B.C., Michener M., Williams M.S. (2012). Outpatient burns: Prevention and care. Am. Fam. Physician.

[20] Palmieri T.L. (2019). Infection Prevention: Unique Aspects of Burn Units. Surg. Infect..

[21] Oryan A., Alemzadeh E., Moshiri A. (2017). Burn wound healing: Present concepts, treatment strategies and future directions. J. Wound Care.

[22] Khansa I., Schoenbrunner A.R., Kraft C.T., Janis J.E. (2019). Silver in Wound Care—Friend or Foe? A Comprehensive Review. Plast. Reconstr. Surg.—Glob. Open.

[23] Negut I., Grumezescu V., Grumezescu A. (2018). Treatment Strategies for Infected Wounds. Molecules.

[24] Poon V.K.M., Burd A. (2004). In vitro cytotoxity of silver: Implication for clinical wound care. Burns.

[25] Abou El-Nour K.M.M., Eftaiha A., Al-Warthan A., Ammar R.A.A. (2010). Synthesis and applications of silver nanoparticles. Arab. J. Chem..

[26] Reis C.P., Neufeld R.J., Veiga F., Ribeirod A.J. (2017). Preparation of drug-loaded polymeric nanoparticles. Nanomedicine in Cancer.

[27] Bastos V., Ferreira de Oliveira J.M.P., Brown D., Johnston H., Malheiro E., Daniel-da-Silva A.L., Duarte I.F., Santos C., Oliveira H. (2016). Corrigendum to “The influence of Citrate or PEG coating on silver nanoparticle toxicity to a human keratinocyte cell line” [Toxicol. Lett. 249 (2016) 29–41]. Toxicol. Lett..

[28] De Matteis V., Cascione M., Toma C., Leporatti S. (2018). Silver Nanoparticles: Synthetic Routes, In Vitro Toxicity and Theranostic Applications for Cancer Disease. Nanomaterials.

[29] Rai M., Deshmukh S.D., Ingle A.P., Gupta I.R., Galdiero M., Galdiero S. (2016). Metal nanoparticles: The protective nanoshield against virus infection. Crit. Rev. Microbiol..

[30] Palza H. (2015). Antimicrobial Polymers with Metal Nanoparticles. Int. J. Mol. Sci..

[31] Liao C., Li Y., Tjong S. (2019). Bactericidal and Cytotoxic Properties of Silver Nanoparticles. Int. J. Mol. Sci..

[32] Lee S., Jun B.-H. (2019). Silver Nanoparticles: Synthesis and Application for Nanomedicine. Int. J. Mol. Sci..

[33] Zhang X.-F., Liu Z.-G., Shen W., Gurunathan S. (2016). Silver Nanoparticles: Synthesis, Characterization, Properties, Applications, and Therapeutic Approaches. Int. J. Mol. Sci..

[34] Cadinoiu A.N., Rata D.M., Daraba O.M., Ichim D.L., Popescu I., Solcan C., Solcan G. (2022). Silver Nanoparticles Biocomposite Films with Antimicrobial Activity: In Vitro and In Vivo Tests. Int. J. Mol. Sci..

[35] Al-Musawi S., Albukhaty S., Al-Karagoly H., Sulaiman G.M., Alwahibi M.S., Dewir Y.H., Soliman D.A., Rizwana H. (2020). Antibacterial Activity of Honey/Chitosan Nanofibers Loaded with Capsaicin and Gold Nanoparticles for Wound Dressing. Molecules.

[36] Mihai M.M., Dima M.B., Dima B., Holban A.M. (2019). Nanomaterials for Wound Healing and Infection Control. Materials.

[37] Bruna T., Maldonado-Bravo F., Jara P., Caro N. (2021). Silver Nanoparticles and Their Antibacterial Applications. Int. J. Mol. Sci..

[38] Nam G., Rangasamy S., Purushothaman B., Song J.M. (2015). The Application of Bactericidal Silver Nanoparticles in Wound Treatment. Nanomater. Nanotechnol..

[39] Wong K.K.Y., Liu X. (2010). Silver nanoparticles—The real “silver bullet” in clinical medicine?. Medchemcomm.

[40] Bercea M., Darie R.N., Nit L.E., Morariu S. (2011). Temperature Responsive Gels Based on Pluronic F127 and Poly (vinyl alcohol). Ind. Eng. Chem. Res..

[41] Moreno E., Schwartz J., Larrañeta E., Nguewa P.A., Sanmartín C., Agüeros M., Irache J.M., Espuelas S. (2014). Thermosensitive hydrogels of poly(methyl vinyl ether-co-maleic anhydride)—Pluronic^®^ F127 copolymers for controlled protein release. Int. J. Pharm..

[42] Dewan M., Sarkar G., Bhowmik M., Das B., Chattoapadhyay A.K., Rana D., Chattopadhyay D. (2017). Effect of gellan gum on the thermogelation property and drug release profile of Poloxamer 407 based ophthalmic formulation. Int. J. Biol. Macromol..

[43] Miastkowska M., Kulawik-Pióro A., Szczurek M. (2020). Nanoemulsion Gel Formulation Optimization for Burn Wounds: Analysis of Rheological and Sensory Properties. Processes.

[44] Parhi R. (2017). Cross-linked hydrogel for pharmaceutical applications: A review. Adv. Pharm. Bull..

[45] Jaquilin P.J.R., Oluwafemi O.S., Thomas S., Oyedeji A.O. (2022). Recent advances in drug delivery nanocarriers incorporated in temperature-sensitive Pluronic F-127–A critical review. J. Drug Deliv. Sci. Technol..

[46] Escobar-Chávez J.J., López-Cervantes M., Naïk A., Kalia Y.N., Quintanar-Guerrero D., Ganem-Quintanar A. (2006). Applications of thermo-reversible pluronic F-127 gels in pharmaceutical formulations. J. Pharm. Pharm. Sci..

[47] Diniz I.M.A., Chen C., Xu X., Ansari S., Zadeh H.H., Marques M.M., Shi S., Moshaverinia A. (2015). Pluronic F-127 hydrogel as a promising scaffold for encapsulation of dental-derived mesenchymal stem cells. J. Mater. Sci. Mater. Med..

[48] Park H.-S., Pham C., Paul E., Padiglione A., Lo C., Cleland H. (2017). Early pathogenic colonisers of acute burn wounds: A retrospective review. Burns.

[49] Iravani S., Korbekandi H., Mirmohammadi S.V., Zolfaghari B. (2014). Synthesis of silver nanoparticles: Chemical, physical and biological methods. Res. Pharm. Sci..

[50] Guilger-Casagrande M., de Lima R. (2019). Synthesis of Silver Nanoparticles Mediated by Fungi: A Review. Front. Bioeng. Biotechnol..

[51] Haider A., Kang I.-K. (2015). Preparation of Silver Nanoparticles and Their Industrial and Biomedical Applications: A Comprehensive Review. Adv. Mater. Sci. Eng..

[52] Abbasi E., Milani M., Fekri Aval S., Kouhi M., Akbarzadeh A., Tayefi Nasrabadi H., Nikasa P., Joo S.W., Hanifehpour Y., Nejati-Koshki K. (2016). Silver nanoparticles: Synthesis methods, bio-applications and properties. Crit. Rev. Microbiol..

[53] Natsuki J. (2015). A Review of Silver Nanoparticles: Synthesis Methods, Properties and Applications. Int. J. Mater. Sci. Appl..

[54] Moreno-Martin G., León-González M.E., Madrid Y. (2018). Simultaneous determination of the size and concentration of AgNPs in water samples by UV–vis spectrophotometry and chemometrics tools. Talanta.

[55] Singh R., Shedbalkar U.U., Wadhwani S.A., Chopade B.A. (2015). Bacteriagenic silver nanoparticles: Synthesis, mechanism, and applications. Appl. Microbiol. Biotechnol..

[56] Patil M.P., Kim G.-D. (2017). Eco-friendly approach for nanoparticles synthesis and mechanism behind antibacterial activity of silver and anticancer activity of gold nanoparticles. Appl. Microbiol. Biotechnol..

[57] Tang S., Zheng J. (2018). Antibacterial Activity of Silver Nanoparticles: Structural Effects. Adv. Healthc. Mater..

[58] Bélteky P., Rónavári A., Zakupszky D., Boka E., Igaz N., Szerencsés B., Pfeiffer I., Vágvölgyi C., Kiricsi M., Kónya Z. (2021). Are Smaller Nanoparticles Always Better? Understanding the Biological Effect of Size-Dependent Silver Nanoparticle Aggregation Under Biorelevant Conditions. Int. J. Nanomed..

[59] Martínez-Castañón G.A., Niño-Martínez N., Martínez-Gutierrez F., Martínez-Mendoza J.R., Ruiz F. (2008). Synthesis and antibacterial activity of silver nanoparticles with different sizes. J. Nanoparticle Res..

[60] Jeong Y., Lim D.W., Choi J. (2014). Assessment of Size-Dependent Antimicrobial and Cytotoxic Properties of Silver Nanoparticles. Adv. Mater. Sci. Eng..

[61] Sonavane G., Tomoda K., Sano A., Ohshima H., Terada H., Makino K. (2008). In vitro permeation of gold nanoparticles through rat skin and rat intestine: Effect of particle size. Colloids Surfaces B Biointerfaces.

[62] Schneider M., Stracke F., Hansen S., Schaefer U.F. (2009). Nanoparticles and their interactions with the dermal barrier. Dermatoendocrinol.

[63] Ezealisiji K.M., Okorie H.N. (2018). Size-dependent skin penetration of silver nanoparticles: Effect of penetration enhancers. Appl. Nanosci..

[64] Tak Y.K., Pal S., Naoghare P.K., Rangasamy S., Song J.M. (2015). Shape-Dependent Skin Penetration of Silver Nanoparticles: Does It Really Matter?. Sci. Rep..

[65] Kraeling M.E.K., Topping V.D., Keltner Z.M., Belgrave K.R., Bailey K.D., Gao X., Yourick J.J. (2018). In vitro percutaneous penetration of silver nanoparticles in pig and human skin. Regul. Toxicol. Pharmacol..

[66] Martínez-Higuera A., Rodríguez-Beas C., Villalobos-Noriega J.M.A., Arizmendi-Grijalva A., Ochoa-Sánchez C., Larios-Rodríguez E., Martínez-Soto J.M., Rodríguez-León E., Ibarra-Zazueta C., Mora-Monroy R. (2021). Hydrogel with silver nanoparticles synthesized by Mimosa tenuiflora for second-degree burns treatment. Sci. Rep..

[67] Larese F.F., D’Agostin F., Crosera M., Adami G., Renzi N., Bovenzi M., Maina G. (2009). Human skin penetration of silver nanoparticles through intact and damaged skin. Toxicology.

[68] Ong W.T.J., Nyam K.L. (2022). Evaluation of silver nanoparticles in cosmeceutical and potential biosafety complications. Saudi J. Biol. Sci..

[69] Bianco C., Visser M.J., Pluut O.A., Svetličić V., Pletikapić G., Jakasa I., Riethmuller C., Adami G., Larese Filon F., Schwegler-Berry D. (2016). Characterization of silver particles in the stratum corneum of healthy subjects and atopic dermatitis patients dermally exposed to a silver-containing garment. Nanotoxicology.

[70] Wang M., Marepally S.K., Vemula P.K., Xu C. (2016). Inorganic Nanoparticles for Transdermal Drug Delivery and Topical Application. Nanoscience in Dermatology.

[71] Kim J.S., Kuk E., Yu K.N., Kim J.-H., Park S.J., Lee H.J., Kim S.H., Park Y.K., Park Y.H., Hwang C.-Y. (2007). Antimicrobial effects of silver nanoparticles. Nanomed. Nanotechnol. Biol. Med..

[72] Ahmadi M., Adibhesami M. (2017). The Effect of Silver Nanoparticles on Wounds Contaminated with Pseudomonas aeruginosa in Mice: An Experimental Study. Iran. J. Pharm. Res. IJPR.

[73] Latifi N.A., Karimi H. (2017). Correlation of occurrence of infection in burn patients. Ann. Burn. Fire Disasters.

[74] Rigo C., Ferroni L., Tocco I., Roman M., Munivrana I., Gardin C., Cairns W., Vindigni V., Azzena B., Barbante C. (2013). Active Silver Nanoparticles for Wound Healing. Int. J. Mol. Sci..

[75] Mota A.H., Prazeres I., Mestre H., Bento-Silva A., Rodrigues M.J., Duarte N., Serra A.T., Bronze M.R., Rijo P., Gaspar M.M. (2021). A Newfangled Collagenase Inhibitor Topical Formulation Based on Ethosomes with *Sambucus nigra* L. Extract. Pharmaceuticals.

[76] Masood N., Ahmed R., Tariq M., Ahmed Z., Masoud M.S., Ali I., Asghar R., Andleeb A., Hasan A. (2019). Silver nanoparticle impregnated chitosan-PEG hydrogel enhances wound healing in diabetes induced rabbits. Int. J. Pharm..

[77] Nguyen T.D., Nguyen T.T., Ly K.L., Tran A.H., Nguyen T.T.N., Vo M.T., Ho H.M., Dang N.T.N., Vo V.T., Nguyen D.H. (2019). In Vivo Study of the Antibacterial Chitosan/Polyvinyl Alcohol Loaded with Silver Nanoparticle Hydrogel for Wound Healing Applications. Int. J. Polym. Sci..

[78] Ahsan A., Farooq M.A. (2019). Therapeutic potential of green synthesized silver nanoparticles loaded PVA hydrogel patches for wound healing. J. Drug Deliv. Sci. Technol..

[79] Xie Y., Liao X., Zhang J., Yang F., Fan Z. (2018). Novel chitosan hydrogels reinforced by silver nanoparticles with ultrahigh mechanical and high antibacterial properties for accelerating wound healing. Int. J. Biol. Macromol..

[80] Badhwar R., Mangla B., Neupane Y.R., Khanna K., Popli H. (2021). Quercetin loaded silver nanoparticles in hydrogel matrices for diabetic wound healing. Nanotechnology.

[81] Faris Taufeq F.Y., Habideen N.H., Rao L.N., Podder P.K., Katas H. (2023). Potential Hemostatic and Wound Healing Effects of Thermoresponsive Wound Dressing Gel Loaded with *Lignosus rhinocerotis* and *Punica granatum* Extracts. Gels.

[82] Shriky B., Kelly A., Isreb M., Babenko M., Mahmoudi N., Rogers S., Shebanova O., Snow T., Gough T. (2020). Pluronic F127 thermosensitive injectable smart hydrogels for controlled drug delivery system development. J. Colloid Interface Sci..

[83] Gioffredi E., Boffito M., Calzone S., Giannitelli S.M., Rainer A., Trombetta M., Mozetic P., Chiono V. (2016). Pluronic F127 Hydrogel Characterization and Biofabrication in Cellularized Constructs for Tissue Engineering Applications. Procedia CIRP.

[84] Zhang K., Lui V.C.H., Chen Y., Lok C.N., Wong K.K.Y. (2020). Delayed application of silver nanoparticles reveals the role of early inflammation in burn wound healing. Sci. Rep..

[85] Stojkovska J., Djurdjevic Z., Jancic I., Bufan B., Milenkovic M., Jankovic R., Miskovic-Stankovic V., Obradovic B. (2018). Comparative in vivo evaluation of novel formulations based on alginate and silver nanoparticles for wound treatments. J. Biomater. Appl..

[86] Yang Z., Nie S., Hsiao W.W., Pam W. (2011). Thermoreversible Pluronic&reg; F127-based hydrogel containing liposomes for the controlled delivery of paclitaxel: In vitro drug release, cell cytotoxicity, and uptake studies. Int. J. Nanomed..

[87] Braiman-Wiksman L., Solomonik I., Spira R., Tennenbaum T. (2007). Novel Insights into Wound Healing Sequence of Events. Toxicol. Pathol..

